# *qPH_1.01_*, a dominant QTL that contributes to plant height and ear height in maize

**DOI:** 10.1002/tpg2.70157

**Published:** 2025-11-20

**Authors:** Chuan Chen, Shaoqi Yang, Ruiyu Zhang, Yuan Tian, Haotian Zhang, Yapeng Wang, Hongwei Zhang, Zhe Wang, Qin Yang

**Affiliations:** ^1^ State Key Laboratory of Crop Stress Resistance and High‐Efficiency Production, College of Agronomy Northwest A&F University Yangling China; ^2^ Hainan Research Institute of Northwest A&F University Sanya China; ^3^ Key Laboratory of Maize Biology and Genetic Breeding in Arid Area of Northwest Region of the Ministry of Agriculture Northwest A&F University Yangling China; ^4^ Institute of Crop Science Chinese Academy of Agricultural Sciences Beijing China

## Abstract

Plant height (PH) is an important agronomic trait closely related to yield and lodging resistance. In this study, bulked segregant analysis coupled with next‐generation sequencing (BSA‐seq) approach was employed to identify quantitative trait loci (QTLs) for PH in a large F_2_ population generated from a cross between the tall maize (*Zea mays* L.) inbred line PH6WC and the short maize inbred line KA3321. Extreme high‐ and low‐PH bulks were constructed by selecting 62 individuals each from the tails of the phenotypic distribution in the 617‐member F_2_ population.  BSA‐seq revealed four PH QTLs, two of which were validated in a random F_2_ population. *qPH_1.01_*, a novel QTL on chromosome 1, showed dominance effect for reducing both PH and ear height (EH). The KA3321 allele of *qPH_1.01_* reduced PH by 8–12 cm and EH by 4–6 cm. Using recombinant‐derived progeny testing strategy, *qPH_1.01_* was fine‐mapped to an interval of approximately 420 kb referring to B73 genome. Transcriptome data showed that *qPH_1.01_* might influence PH and EH by regulating expression of hormone‐related genes. The dominant QTL discovered in this study may facilitate the breeding of dwarf and high‐density maize varieties.

## Core Ideas

- We identified a novel dominant quantitative trait locus (QTL), *qPH_1.01_*, on maize chromosome 1, with allele from KA3321 reducing plant height by 8–12 cm and ear height by 4–6 cm, respectively.
- Using recombinant‐derived progeny testing strategy, we narrowed down *qPH_1.01_* to a 420‐kb region referring to B73 genome.
- RNA‐seq results suggested that *qPH_1.01_* might influence plant height and ear height by regulating expression of hormone‐related genes.
- *qPH_1.01_* could be beneficial for breeding dwarf and high‐density maize varieties.

## Plain Language Summary

Plant height (PH) is an important agronomic trait closely associated with yield and lodging resistance. Using bulked segregant analysis coupled with next‐generation sequencing (BSA‐seq), we identified four quantitative trait loci (QTLs) that contribute to plant height in maize. QTL validation in a random F_2_ population showed that *qPH_1.01_* was a dominant QTL for both PH and EH, with the allele from KA3321 reducing PH by 8–12 cm and EH by 4–6 cm respectively. Through years of fine‐mapping, we narrowed down *qPH_1.01_* to a 420‐kb genomic region referring to B73 genome. RNA‐seq results suggested that *qPH_1.01_* might influence PH and EH by regulating expression of hormone‐related genes. The tightly linked molecular markers developed in the *qPH_1.01_* region could facilitate map‐based cloning and marker‐assisted selection in maize dwarf breeding.

**Abbreviations**
AD: allele depth
BR: brassinosteroid
BSA‐seq: bulked segregant analysis coupled with next‐generation sequencing
CTAB: cetyltrimethylammonium bromide
DEG: differentially expressed gene
EH: ear height
GA: gibberellin
GO: Gene Ontology
GWAS: genome‐wide association studies
KASP: kompetitive allele‐specific PCR
MAS: marker‐assisted selection
PH: plant height
QTL: quantitative trait locus
RILs: recombinant inbred lines
SNP: single nucleotide polymorphism

## 1 INTRODUCTION

Maize (*Zea mays* L.) is one of the most important food crops worldwide. Maize plant height (PH) is often associated with ear height (EH), and both are closely related to lodging resistance and yield (W. Wang et al., 2023).

Maize PH is determined by the number of nodes and the length of internodes in the stem, and EH is determined by the number of nodes and the length of internodes below the ear (Tang et al., 2007). A wide range of studies have shown that PH and EH are complex quantitative traits controlled by a large number of genes. Quantitative trait locus (QTL) mapping and genome‐wide association studies (GWAS) have been extensively used to identify QTLs associated with PH and EH in maize. According to the MaizeGDB database (http://www.maizegdb.org/), 283 QTLs for PH and 46 for EH have been documented, distributed across all 10 maize chromosomes. Among them, 15.68–20.40 Mb of chromosome 2, 217.07–224.60 Mb of chromosome 3, and 23.11–94.28 Mb of chromosome 9 were the hot spots for colocalized QTLs of PH and EH (Ma et al., 2021). GWAS also identified many single nucleotide polymorphisms (SNPs) associated with maize PH and EH. Hot spots for colocalized GWAS hits of PH and EH were identified at 4.38–8.68 Mb of chromosome 1 and 237.95–244.09 Mb of chromosome 2 (Ma et al., 2021). Among the genes reported to be associated with maize PH and EH, most of them are related to hormone, including gibberellin (GA), brassinosteroids (BRs), auxin, and ethylene (W. Wang et al., 2023). *Dwarf3* (*D3*), *ZmGA3ox2*, and *An2* affect maize PH by regulating GA biosynthesis pathway (Harris et al., 2005; Teng et al., 2013; Winkler & Helentjaris, 1995)*. na2* reduces downstream BR metabolites by accumulating the DWF1 substrate 24‐methylene cholesterol, ultimately leading to plant dwarfing (Best et al., 2016). *Br2* and *ZmTE1* regulate maize PH by affecting auxin polar transport and signal transduction (Multani et al., 2003; F. Wang et al., 2022). By catalyzing the synthesis of ethylene precursor ACC, *zmacs7* increases the contents of ACC and ethylene in plants, resulting in dwarfing phenotype (H. Li et al., 2020). Although many genes related to PH and EH have been characterized, some genes exhibit various developmental defects, such as deformed flowers and abnormal ears, limiting their potential application in breeding programs (Jafari et al., 2024).

Bulked segregant analysis (BSA) is a time‐ and cost‐effective QTL mapping method that shows great potential when combined with high‐throughput sequencing technology (Z. Li & Xu, 2022; Liu et al., 2012; Schneeberger et al., 2009). BSA selects only individuals with extreme phenotypes to form a mixed sample pool for genotyping, thereby reducing costs, simplifying analysis, and enhancing mapping resolution. Several algorithms for BSA have been reported and widely applied, such as G‐statistic (Magwene et al., 2011) and ΔSNP‐index (Abe et al., 2012). Bulked segregant analysis coupled with next‐generation sequencing (BSA‐seq) technology has been proven to be successful for rapidly identifying the chromosome region harboring genes/QTLs of interest in numerous crop species (Jia et al., 2023; Lei et al., 2020; C. Li et al., 2024; Takagi et al., 2015; Xin et al., 2020).

Here, we employed BSA‐seq to detect PH QTLs in an F_2_ mapping population derived from the cross between maize inbred lines PH6WC and KA3321. A major dominant QTL, *qPH_1.01_*, on chromosome 1 that reduces both PH and EH was narrowed down to an interval of approximately 420 kb. This study may provide the basis for cloning the quantitative trait gene underlying this dominant QTL.

## 2 MATERIALS AND METHODS

### 2.1 Plant materials

The initial QTL mapping population comprised 617 F_2_ families, derived from a cross between the tall maize inbred line PH6WC and the short maize inbred line KA3321. PH6WC is the maternal parent of Xianyu335, a leading hybrid variety in China. KA3321 is a self‐selected maternal line derived by crossing Huanong 887 and Huamei No. 1, and it exhibits a short phenotype in PH and EH. For initial QTL mapping, field experiments were performed during the summer of 2021 in Yangling of China (34.28°N, 108.06°E); in May 2021, plots were planted at 3 m in length with 0.6 m between rows. A total of 617 F_2_ plants were acquired. After measuring individual PH at maturity, the top and bottom 10% of plant materials based on height were selected for BSA.

### 2.2 Field experiments and trait measurement

Field experiments were performed during the summers of 2020–2023 in Yangling (34.28°N, 108.06°E) of Shaanxi province and winters of 2020–2022 in Sanya (18.24°N, 109.51°E) of Hainan province. In Yangling, plots were planted at 3 m in length with 0.6 m between rows. In Sanya, plots were planted at 2.5 m in length with 0.6 m between rows. The PH and EH of each plant were measured at the mature stage. PH was determined as the distance from ground to the top of tassel (cm), and EH was determined as the distance from ground to the first ear node (cm).

### 2.3 DNA sequencing and genotyping

DNA was extracted from fresh leaves of the 617 F_2_ plants by cetyltrimethylammonium bromide (CTAB) method. DNA from the top 10% of individuals with extreme phenotypes was pooled in equal amounts to construct two separate pools, a high‐value pool (H‐pool) and a low‐value pool (L‐pool), each comprising 62 individuals. DNA was sent to Shenzhen BGI Genomics Co., Ltd. for sequencing with DNBseq Platform, generating a total of 75 Gbase of sequence data from each pool. The reads mapping and SNP calling procedure were performed as described by H. Zhang et al. (2019). After quality control, clean reads were mapped to the maize reference genome (Zm‐B73‐REFERENCE‐NAM‐5.0) by the BWA software (0.7.17) (http://bio‐bwa.sourceforge.net/bwa.shtml). SAMtools was used to convert sam to bam and make index file (http://samtools.github.io/). The Picard software was employed to fix mates, sort read groups, and remove PCR duplicates from the bam files (https://broadinstitute.github.io/picard/). SNPs and insertion/deletion (InDel) mutations were identified using the GATK (4.1.9.0) software (McKenna et al., 2010).

**Core Ideas**

- We identified a novel dominant quantitative trait locus (QTL), *qPH_1.01_*, on maize chromosome 1, with allele from KA3321 reducing plant height by 8–12 cm and ear height by 4–6 cm, respectively.
- Using recombinant‐derived progeny testing strategy, we narrowed down *qPH_1.01_* to a 420‐kb region referring to B73 genome.
- RNA‐seq results suggested that *qPH_1.01_* might influence plant height and ear height by regulating expression of hormone‐related genes.
- *qPH_1.01_* could be beneficial for breeding dwarf and high‐density maize varieties.

### 2.4 Calculation of ΔSNP‐index, G‐statistic, and sSNP/totalSNP ratio

Given the limitation of parental genome resequencing data, PyBSAseq was used to calculate ΔSNP‐index, G‐statistic, and sSNP/totalSNP ratio. The sliding window was set with a size of 2,000,000 base pairs and an incremental step of 10,000 base pairs. For the thresholds of ΔSNP‐index and G‐statistic: for each SNP in the dataset, simulated allele depth (AD) values (smADREF1/smADALT1 for bulk 1 and smADREF2/smADALT2 for bulk 2) were generated. Using these simulated AD values, ΔSNP‐index and G‐statistic for each SNP were calculated. This simulation process was repeated 10,000 times. The threshold for ΔSNP‐index was determined as the 99% confidence interval of the 10,000 simulated ΔSNP‐index values, while the threshold for G‐statistic was set as the 99.5th percentile of the 10,000 simulated G‐statistic values. The SNP index (allele frequency) of a given SNP was calculated by dividing the alternative read count by the total read count (reference read count + alternative read count) in each bulk (Abe et al., 2012). ΔSNP‐index is the difference of SNP‐index between H‐pool and L‐pool as described previously (Takagi et al., 2013).

$$\begin{array}{ccc} \Delta \mathrm{SNP}-index & = & \frac{\mathrm{Allele}\;\mathrm{Depth}\left(H-\mathrm{pool}\right)}{\mathrm{Total}\;\mathrm{Depth}\;\mathrm{of}\;H-\mathrm{pool}}\\ & & \;-\;\frac{\mathrm{Allele}\;\mathrm{Depth}\left(L-\mathrm{pool}\right)}{\mathrm{Total}\;\mathrm{Depth}\;\mathrm{of}\;L-\mathrm{pool}}. \end{array}$$

The G‐statistic, a log‐likelihood ratio test statistic evaluating the significance of allele frequency differences between the two bulks, was calculated as described by Magwene et al. (2011) and averaged across neighboring SNPs:

$$G=\sum_{i}O_{i}\times \ln\left(O_{i}\times E_{i}\right).$$

The significant SNPs (sSNPs) associated with the trait were identified via Fisher's exact test, and then the ratio of the sSNPs to total SNPs (sSNP/total SNP) in a chromosomal interval was used to detect the genomic regions that condition the trait of interest.

### 2.5 Molecular marker development and genotyping

Genomic DNA was extracted from fresh leaves using CTAB method. In this study, an allele‐specific quantitative PCR‐based genotyping assay, an enhanced system based on the kompetitive allele‐specific PCR (KASP) principle, was utilized (Beijing JasonGen Biological Technology Co., Ltd). KASP markers were designed in the 5.5–6.5 Mb region of chromosome 1 according to SNPs detected from mapping data of BSA‐seq. For each SNP, 500 bp upstream and downstream flanking genomic sequences of SNPs were used for the design of KASP primers (design criteria: length of amplified fragment about 150 bp, GC content 40%–60%, forward primer TM value 61–63°C, reverse primer TM value 63–65°C, length 19–25 bp). The specificity of primers was examined by BLAST at MaizeGDB (https://maizegdb.org/) and sent to the company for synthesis (Sangon Biotech [Shanghai] Co., Ltd.). For each pair of KASP markers, we have validated the genotyping accuracy by comparing the sanger sequencing results with KASP genotyping results from at least 16 samples including the two parental lines and the F_1_ plant. Only the primers showing consistent results were used for fine‐mapping. All KASP primers are listed in Table S1. These markers were used to genotype the segregated population.

### 2.6 Fine mapping of *qPH_1.01_*

In order to fine‐map *qPH_1.01_*, a major QTL on chromosome 1, we identified 37 recombinant F_2_ plants, which showed a chromosomal recombination in the major QTL region. The 37 F_2:3_ families were planted in the Hainan winter nursery in Sanya during 2021/2022 for self‐pollination to produce the F_3:4_ families. In 2022, eight F_3:4_ families were planted in Hainan for fine‐mapping. Among them, seven families were homozygous fixed F_3:4_ progenies, while the remaining one was a segregating F_3:4_ progeny. To further narrow down the QTL region, new recombinants were screened based on the newly mapped region. Ten derived F_4:5_ progeny lines were then evaluated in Yangling during the 2023 growing season. Number of progeny lines of the recombinant families ranged from 50 to 300, and the evaluation was conducted on individual plants.

### 2.7 Transcriptome analysis

For RNA‐seq analysis, we collected developing nodes and stalks at 40 days from a pair of heterogeneous inbred family‐derived near‐isogenic lines (NILs), NIL‐*qPH_1.01PH6WC_* and NIL‐*qPH_1.01KA3321_*, which segregated for *qPH_1.01_*. Stem nodes were sampled from the internode between nodes 8 and 9 (6‐8 mm); stalk was sampled from the spike node (node 8) adjacent to the internode (6–8 mm). Six plants of each genotype were pooled as one biological replicate. Two biological replicates were collected per genotype for each tissue type. The samples were sent to Yazhou Bay Seed Laboratory for mRNA extraction and sequencing. RNA quality was evaluated using an Agilent Bioanalyzer System 2100 (Agilent Technologies, Palo Alto, CA, USA), and samples with an RNA integrity number > 7.5 were used for cDNA libraries construction. All cDNA libraries were sequenced on an Illumina sequencing platform (NovaSeq 6000) by paired‐end (2 × 150 bp) method. Each library was sequenced to obtain an average of ∼25.3 million paired‐end raw reads (Supplemental Table S2). Raw reads were filtered by Trimmomatic (Bolger et al., 2014). A total of 53.39‐Gb clean reads were generated and mapped to the B73 reference genome (Zm‐B73‐REFERENCE‐NAM‐5.0) using the Hisat2 software (Kim et al., 2019). FeaturesCounts was used to count the reads mapped in the annotated gene model. Transcripts per million values of all datasets were extracted using StringTie (Pertea et al., 2016). Differentially expressed genes (DEGs) were determined using the R package DESeq2 [| log2(fold change) | ≥ 1, *p* adj < 0.05]. Gene Ontology (GO) terms were enriched using TBtools (Chen et al., 2023). GO terms with corrected *p*‐value < 0.05 were considered to be significantly enriched.

### 2.8 Statistical data analysis

Microsoft Excel was employed to compute the mean and standard error (SE) for statistical significance analyses. Significant differences were determined by Student's *t*‐test.

## 3 RESULTS

### 3.1 Phenotypic evaluation of parental lines

**FIGURE 1:**
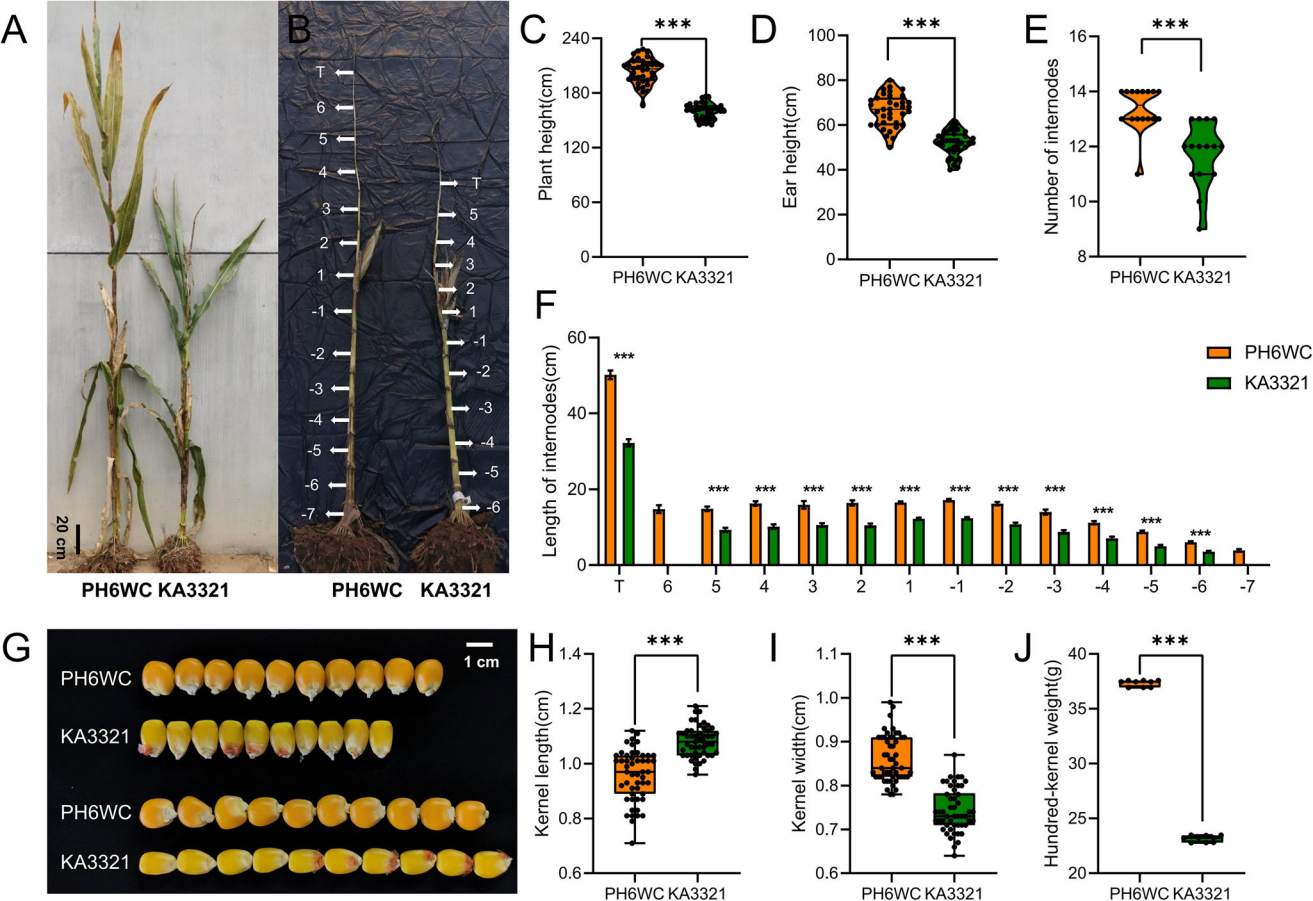
PH6WC and KA3321 were significantly different in plant height, ear height, the number of nodes, the length of internodes, kernel size, and hundred‐kernel weight. (A,B) PH6WC and KA3321 field phenotypes at the mature stage. The nodes are indicated by white arrows. Scale bar = 20 cm. (C,D) Plant height (PH) and Ear height (EH) of PH6WC and KA3321 at the mature stage (***p < 0.001). (E) Number of internodes of PH6WC and KA3321 at the mature stage (***p < 0.001). (F) Comparison of the length of the tassel and internodes of PH6WC and KA3321. Asterisks indicate significant differences between PH6WC and KA3321 (***p < 0.001). The internodes above the ear are labeled 1, 2, 3, 4, 5, and 6. The internodes below the ear are labeled −1, −2, −3, −4, −5, −6, and −7. (G) Kernel size of PH6WC and KA3321. Scale bar = 1 cm. (H,I) The kernel size of PH6WC and KA3321 (***p < 0.001). (J) Mature hundred‐kernel weight statistical in PH6WC and KA3321 (***p < 0.001). T, tassel.

A genetic population was constructed with PH6WC as the maternal parent and KA3321 as the paternal parent, exhibiting distinct differences with respect to PH and EH (Figure 1A). The PH and EH of PH6WC were 203.92 ± 14.07 cm and 66.36 ± 7.03 cm, respectively. The PH and EH of KA3321 were 159.89 ± 8.37 cm and 51.86 ± 5.46 cm, respectively. Both PH and EH showed significant differences between PH6WC and KA3321 (Figure 1C,D). There were also significant differences in the number of internodes between PH6WC and KA3321 (Figure 1B,E). The number of internodes of PH6WC was 13.38 ± 0.78, the number of internodes above ear was 4.50 ± 0.87, and the number of internodes below ear was 6.88 ± 0.48. The number of internodes of KA3321 was 11.73 ± 1.12, the number of internodes above ear was 4.00 ± 0.82, and the number of internodes below ear was 5.73 ± 0.93. We labeled the internodes below the ear from −1 to −7, and those above the ear from 1 to 6, as shown in Figure 1B. All internodes of PH6WC were significantly longer than KA3321 (Figure 1F). Besides, the kernel length of KA3321 was significantly lower than PH6WC, while the kernel width of KA3321 was significantly longer than PH6WC (Figure 1G,H,I), and the hundred‐grain weight of KA3321was significantly lower than PH6WC (Figure 1J).

### 3.2 QTL mapping of PH by BSA

**FIGURE 2:**
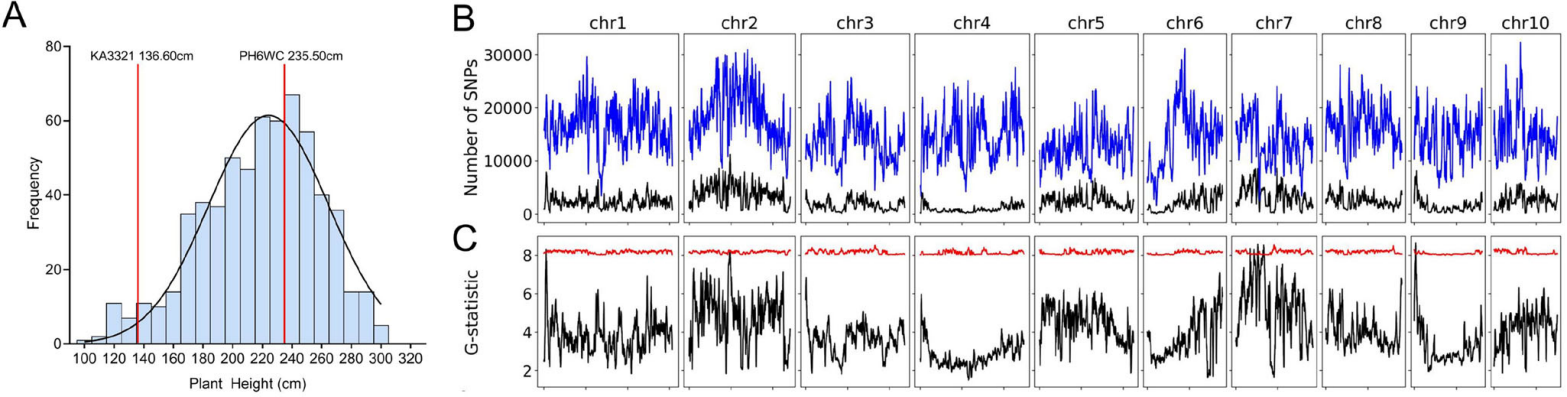
Bulked segregant analysis coupled with next‐generation sequencing (BSA‐seq) data analysis results. (A) Frequency distribution of plant height (PH) in F_2_ segregation population. (B) Genomic distributions of significant single nucleotide polymorphisms (SNPs) (black) and total SNPs (blue). (C) Identification of SNPs associated with PH using G‐statistics method.

**FIGURE 3:**
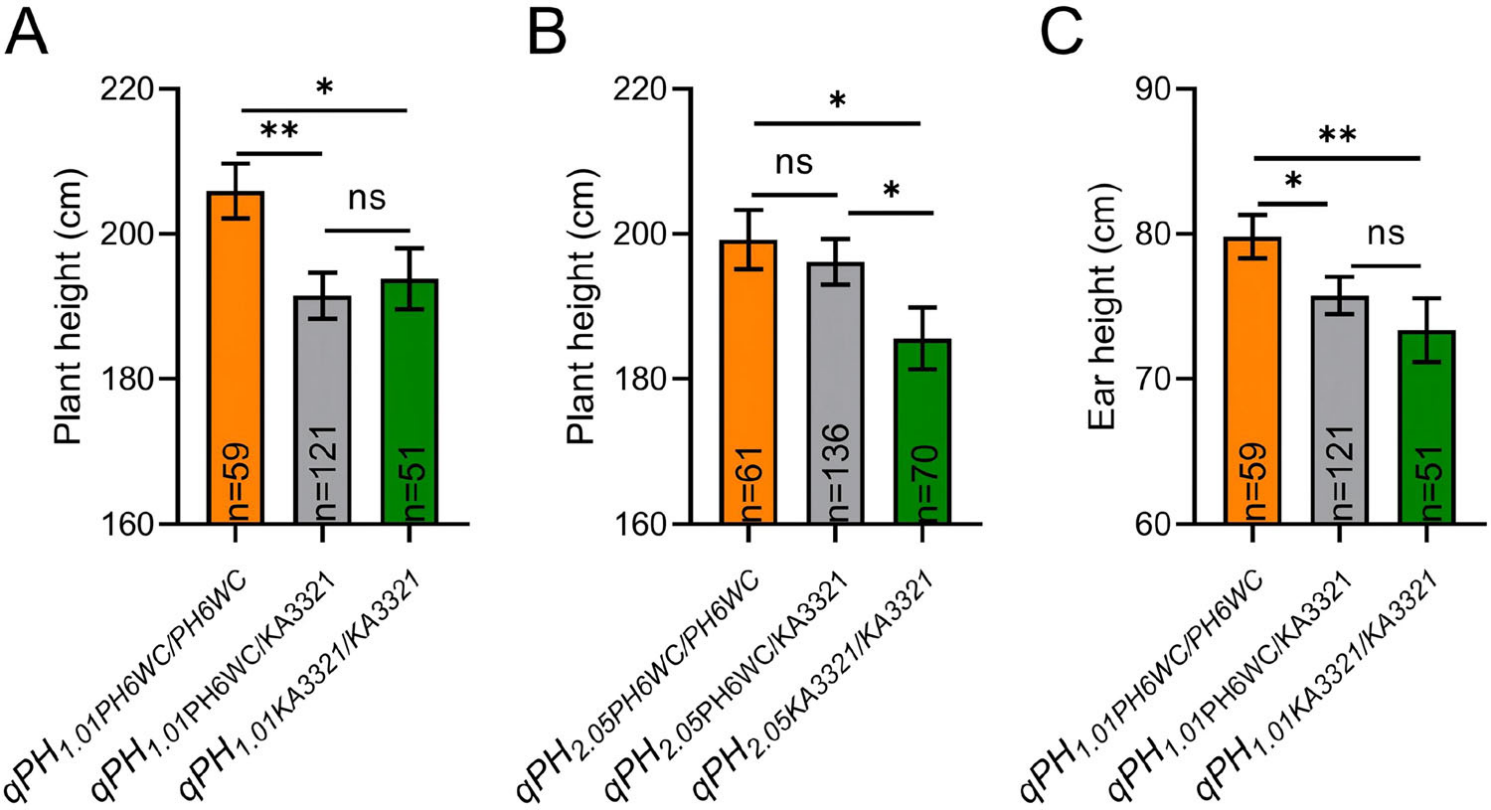
*qPH_1.01_* and *qPH_2.05_* effect validation. Plant height (PH) and ear height (EH) effect validation of *qPH_1.01_* and *qPH_2.05_* locus. A total of 300 individual plants of F_2_ population were randomly selected for genotyping at Ks_5.5 and Ks_97.2 markers to verify *qPH_1.01_* PH effect (A), *qPH_2.05_* PH effect (B), and *qPH_1.01_* EH effect (C). The mean value of PH and EH was depicted for the three genotypes: homozygotes PH6WC/PH6WC, heterozygotes PH6WC/KA3321, and homozygotes KA3321/KA3321 (ns means no significant difference, *p < 0.05, **p < 0.01).

The PH range among the 617 individuals was extensive and conforms to a normal distribution pattern (Figure 2A). Two DNA pools mixed with the highest and lowest PH individuals (n = 62 for each pool) were subjected to paired‐end sequencing. The results of SNP‐calling showed that a total of 16,165,522 SNPs were detected on each chromosome including 2,441,821 significant SNPs (Figure 2B). The average allele depths of the high and low pools were 33.96 × and 32.99 ×, respectively. Using PyBSAseq that combined with three different algorithms, we chose the G‐statistic with 2‐Mb sliding window and 10 kb step size, and four significant signal peaks were identified among chromosomes 1,2,7,9 (G value > 10), suggesting that the region may contain three important QTLs associated with PH. The results from the other two algorithms (ΔSNP‐index and sSNP/totalSNP) were consistent with those from the G‐statistic method. However, the ΔSNP‐index values did not reach the threshold, and the QTL interval identified by the sSNP/totalSNP algorithm spanned several excessively large chromosomal regions (Figure S1). Therefore, the subsequent analysis primarily focused on the intervals derived from the G‐statistic. The physical intervals of these QTLs were 2.08 Mb (from 4.77 to 6.85 Mb), 1.01 Mb (from 96.62 to 97.62 Mb), 35.87 Mb (35.14 to 70.01 Mb) and 2.18 Mb (from 2.65 to 4.83 Mb), respectively, and we named them *qPH_1.01_*, *qPH_2.05_*, *qPH_7.02_*, and *qPH_9.01_*, respectively (Figure 2C; Table 1). Due to the large genomic interval of *qPH_7.02_* and the lack of suitable markers for confirming *qPH_9.01_*, further fine‐mapping efforts were focused primarily on *qPH_1.01_* and *qPH_2.05_*. Effect validation in a random F_2_ population showed that *qPH_1.01_* could decrease PH by 12 cm and *qPH_2.05_* could decrease PH by 14 cm (Figure 3A,B). There was a significant difference in PH between *qPH_1.01PH6WC/KA3321_* heterozygotes and *qPH_1.01PH6WC/PH6WC_* homozygotes, but not between *qPH_1.01PH6WC/KA3321_* heterozygotes and *qPH_1.01KA3321/KA3321_* homozygotes (Figure 3A). These findings implied that *qPH_1.01_* largely acted as a dominant QTL. There was a significant difference in PH between *qPH_2.05PH6WC/KA3321_* heterozygotes and *qPH_2.05KA3321/KA3321_* homozygotes, but not between *qPH_2.05PH6WC/KA3321_* heterozygotes and *qPH_2.05PH6WC/PH6WC_* homozygotes (Figure 3B). These findings implied that *qPH_2.05_* largely acted as a recessive QTL. Effect validation in the F_2_ population showed that *qPH_1.01_* could also decrease EH by 6 cm (Figure 3C). The alleles for decreasing PH and EH were derived from the parent “KA3321.”

**TABLE 1:** Bulked segregant analysis coupled with next‐generation sequencing (BSA‐seq) sequencing location results.

Chromosome	Name	Start/bp	End/bp	Bin
1	*qPH_1.01_*	4,770,076	6,854,877	bin1.01
2	*qPH_2.05_*	97,620,009	98,630,341	bin2.05
7	*qPH_7.02_*	34,140,001	70,01,001	bin7.02
9	*qPH_9.01_*	4,830,181	5,040,135	bin9.01

### 3.3 Fine mapping of *qPH_1.01_*

**FIGURE 4:**
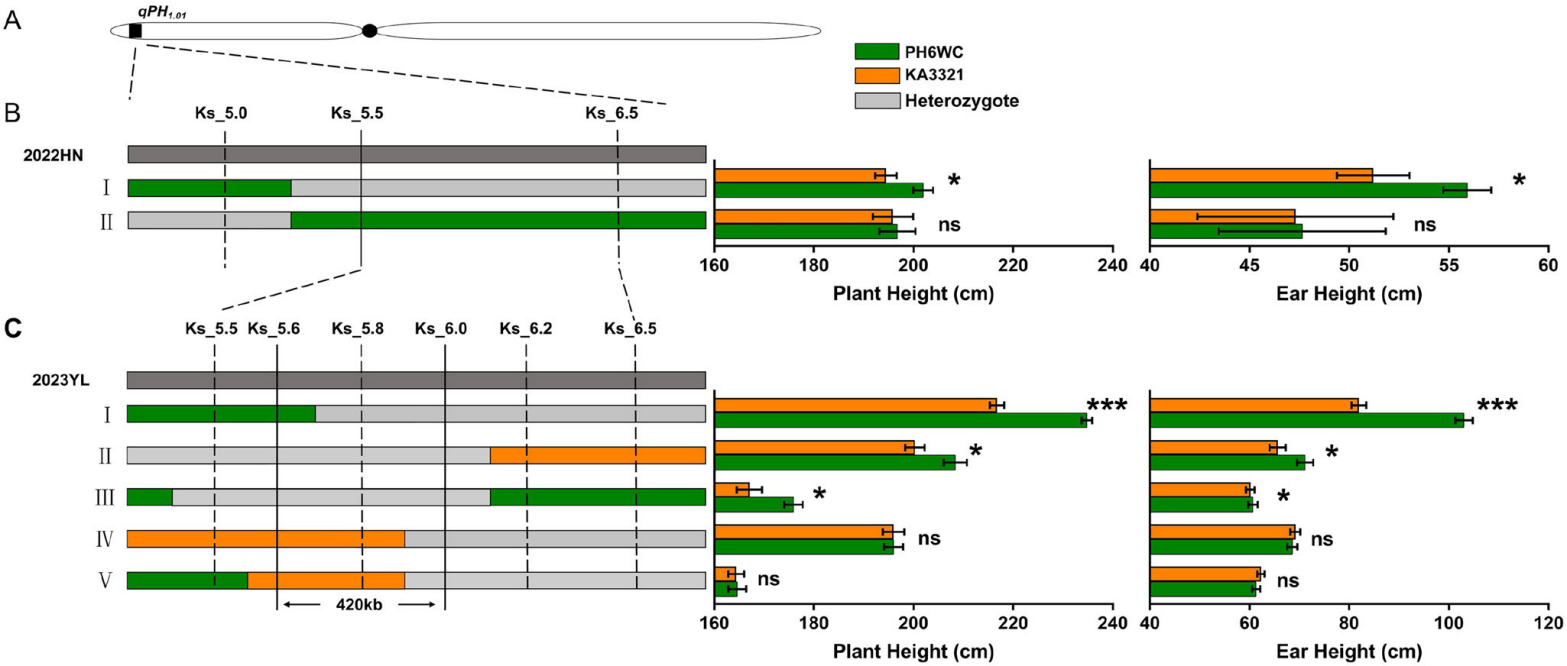
Fine‐mapping of the major quantitative trait locus (QTL) *qPH_1.01_* in maize. Location of the *qPH_1.01_* region in bin1.01 of maize chromosome 1 (A). For each recombinant type, the chromosomal composition at *qPH_1.01_* is depicted as green, yellow, or gray rectangles, corresponding to homozygous PH6WC/PH6WC, homozygous KA3321/KA3321, and heterozygous PH6WC/KA3321, respectively. Markers from heterozygous PH6WC/KA3321 segment were used to genotype all progeny from a given recombinant type. The plant height (PH) and ear height (EH) were calculated for each of the homozygote PH6WC/PH6WC, and homozygote KA3321/KA3321 progeny. A significant difference (*p* < 0.05) between homozygote PH6WC/PH6WC and homozygote KA3321/KA3321 indicated the presence of *qPH_1.01_* in the donor region, and the lack of a significant difference (*p* > 0.05) between these two homozygous genotypes suggested the absence of *qPH_1.01_* in the donor region. Based on the results, we determined the candidate region was the right of Ks_5.0 (B), and narrowed *qPH_1.01_* to 420‐kb genomic region between kompetitive allele‐specific PCR (KASP) markers Ks_5.6 and Ks_6.0 (C).

Based on the initial QTL analysis, two flanking markers (Ks_5.0 and Ks_6.5) and one internal marker (Ks_6.0) within the *qPH_1.01_* region were used to screen the F_2:3_ population. Two types of recombinants were detected with crossover breakpoints within the *qPH_1.01_* region. Subsequently, we evaluated 1800 individuals from the resulting F_3:4_ families in Hainan to test for PH effect and EH effect in the field in 2022 winter. For each F_3:4_ family, markers located in the heterozygous segment were selected to genotype all individuals, thus dividing the F_3:4_ family into three genotypes: PH6WC homozygote (PH6WC/PH6WC), heterozygote (PH6WC/KA3321), and KA3321 homozygote (KA3321/KA3321). A significant (or nonsignificant) difference in PH and EH between PH6WC and KA3321 homozygotes indicated the presence (or absence) of the PH QTL in the heterozygote segment. The genotypes in the *qPH_1.01_* interval and the phenotypes of the progeny families derived from these recombinants are shown in Figure 4B. As shown, type I recombinant inbred lines (RILs) revealed a significant difference (*p* < 0.05) between PH6WC and KA3321 homozygotes, suggesting the presence of *qPH_1.01_* in the heterozygote segment. The type II exhibited no significant differences (*p* > 0.05) between PH6WC and KA3321 homozygotes, suggesting the absence of *qPH_1.01_* in the heterozygote segment. Based on the results, we determined the left boundary of the candidate region was the right of Ks_5.0. Subsequently, we screened recombinants in the F_3:4_ population and identified six types of recombinants. The six types of recombinants were self‐pollinated to produce 10 F_5_ families (2800 individuals) that were planted in Yangling in 2023 summer. As shown in Figure 4C, types I–III recombinant RILs revealed a significant difference (*p *< 0.05) between PH6WC and KA3321 homozygotes, suggesting the presence of *qPH_1.01_* in the heterozygote segment. The remaining types (IV–VI) exhibited no significant differences (*p *> 0.05) between PH6WC and KA3321 homozygotes, suggesting the absence of *qPH_1.01_* in the heterozygote segment. Thus, we narrowed down *qPH_1.01_* to a 420‐kb genomic region between KASP markers Ks_5.6 and Ks_6.0. Effect validation demonstrated that *qPH_1.01_* was a dominant locus for reducing PH and EH. Therefore, during the fine‐mapping process, we also compared the differences in PH and EH between homozygous PH6WC/PH6WC and KA3321/KA3321 genotypes with the heterozygous PH6WC/KA3321 genotype. The results indicated that *qPH_1.01_* indeed acted as a dominant locus for reducing PH and EH, with stable genetic effects across different generations (Table S3).

### 3.4 Transcriptomic analysis of the NILs

**FIGURE 5:**
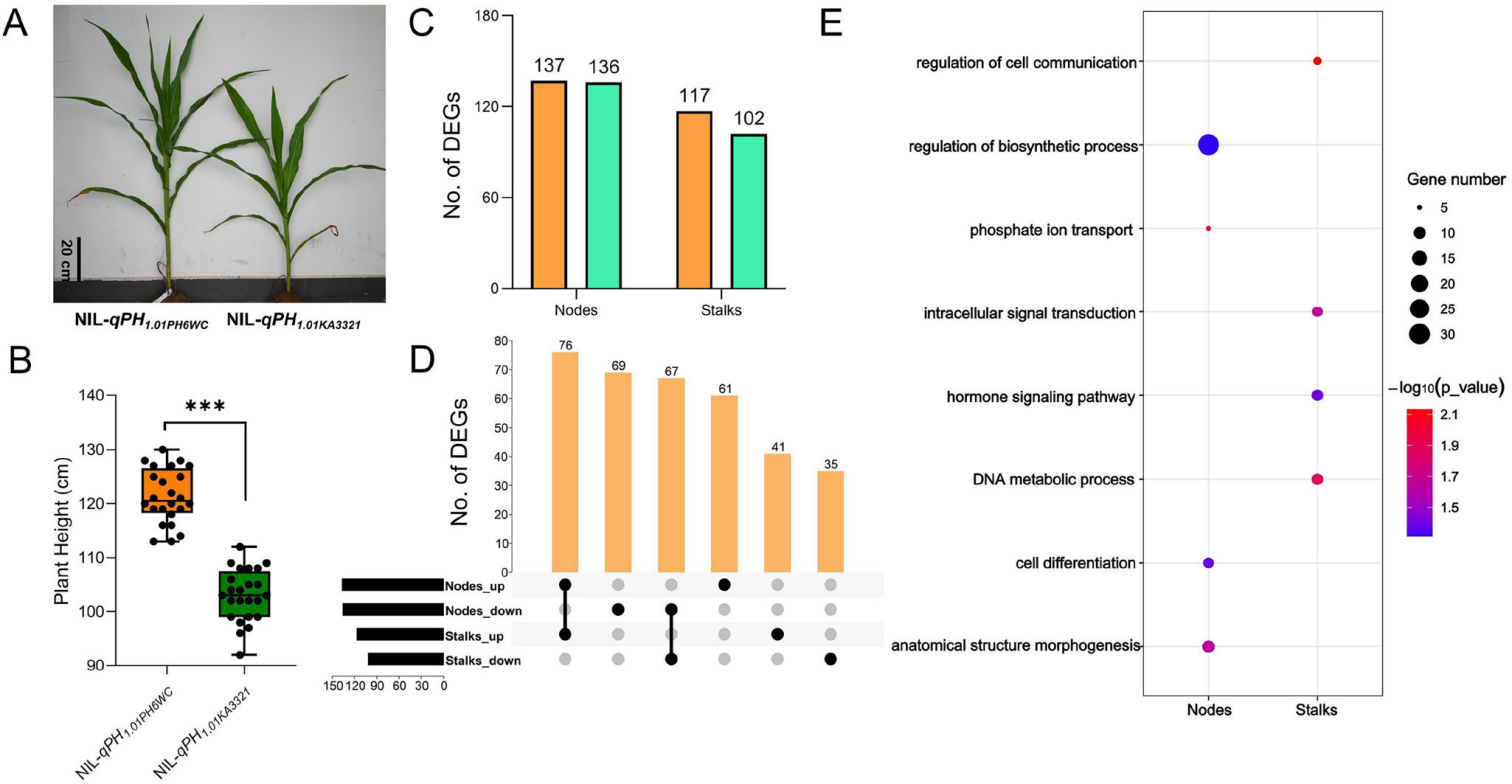
Transcriptomic analysis of NIL‐*qPH_1.01PH6WC_* and NIL‐*qPH_1.01KA3321_*. (A) NIL‐*qPH_1.01PH6WC_* and NIL‐*qPH_1.01KA3321_* field phenotypes at the V9 stage. Scale bar = 20 cm. (B) Plant height (PH) of NIL‐*qPH_1.01PH6WC_* and NIL‐*qPH_1.01KA3321_* at the V9 stage. (***p < 0.001). (C) Number of differentially expressed genes (DEGs) identified in NIL‐*qPH_1.01PH6WC_* and NIL‐*qPH_1.01KA3321_* at nodes and stalks, respectively. (D) Upset plot of the number of shared and specific DEGs of node and stalk identified in NIL‐*qPH_1.01PH6WC_* and NIL‐*qPH_1.01KA3321_*. (E) Gene Ontology (GO)‐term enrichment of the DEGs in nodes and stalks detected at 40 days (V9 stage stage).

**FIGURE 6:**
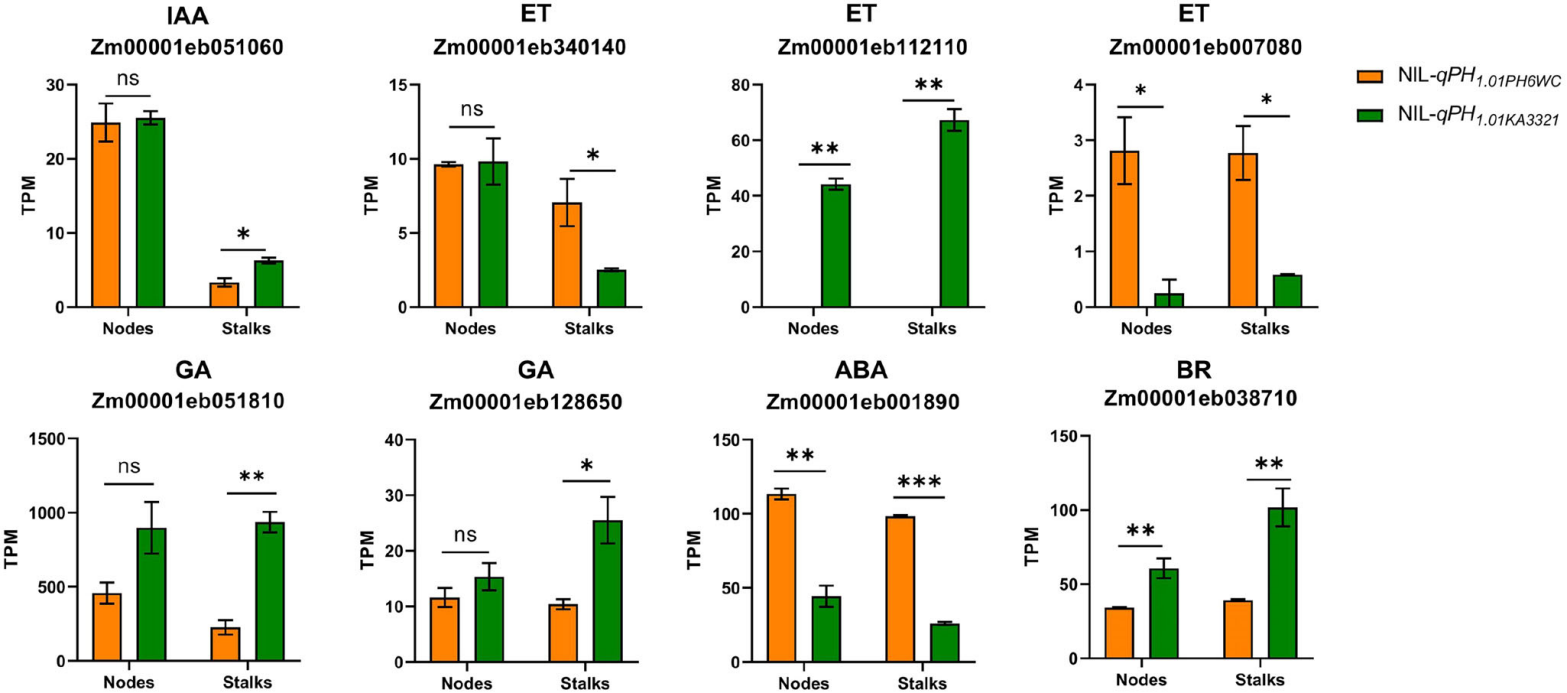
Gene Ontology (GO)‐term enriched differentially expressed genes (DEGs) in NIL‐*qPH_1.01PH6WC_* and NIL‐*qPH_1.01KA3321_* in terms of hormone signaling pathway. Among them, IAA, ET, GA, ABA, and BR represent auxin, ethylene, gibberellin, abscisic acid and brassinosteroids, respectively (ns means no significant difference, * *p* < 0.05, ** *p* < 0.01, *** *p* < 0.001).

To elucidate the molecular mechanism underlying the reduced PH and EH in NIL‐*qPH_1.01KA3321_*, samples from developing nodes and stalks at 40 days of a pair of near‐isogenic lines NIL‐*qPH_1.01PH6WC_* and NIL‐*qPH_1.01KA3321_* were collected and sequenced. RNA‐Seq analysis was performed with two biological replications. The average PH of NIL‐*qPH_1.01PH6WC_* was 121.33 ± 4.90 cm and the average PH of NIL‐*qPH_1.01KA3321_* was 103.00 ± 4.70 cm (Figure 5A,B). In nodes samples, 137 genes were upregulated and 136 downregulated in NIL‐*qPH_1.01PH6WC_* compared with NIL‐*qPH_1.01KA3321_* (Figure 5C; Table S4). In stalks samples, 117 genes were upregulated and 102 downregulated in NIL‐*qPH_1.01PH6WC_* compared with NIL‐*qPH_1.01KA3321_* (Figure 5C; Table S5). Among them, 76 genes were upregulated and 67 downregulated in NIL‐*qPH_1.01PH6WC_* compared with NIL‐*qPH_1.01KA3321_* in both the nodes and stalks (Figure 5D). GO enrichment analysis showed that the DEGs of nodes were enriched in the regulation of biosynthetic process, phosphate ion transport, cell differentiation, and anatomical structure morphogenesis (Figure 5E; Table S6). The GO enrichment of stalks was enriched in regulation of cell communication, intracellular signal transduction, hormone signaling pathway, and DNA metabolic process (Figure 5E; Table S7). In the DEGs labeled with term of hormone signaling pathway, two genes related to ABA were upregulated in NIL‐*qPH_1.01PH6WC_* in both the nodes and stalks; however, genes related to GA and BR were upregulated in NIL‐*qPH_1.01KA3321_* in both the nodes and stalks (Figure 6).

## 4 DISCUSSION

### 4.1 Analysis of *qPH_1.01_* and comparison with previous studies

With the updating of sequencing technology and the development of molecular marker technology, many QTLs related to PH have been identified so far. Some reports on the cloning of PH QTL loci have also appeared, among which the number of QTLs on chromosome 1 is relatively high. Lima et al. (2006) localized the primary plant height QTL *qPH1a* (*bnlg1178‐bnlg1953*) to bin1.02 (chromosome 1) using 256 F_2:3_ families from a tropical maize population. Tang et al. (2007) employed SSR markers to map two PH QTLs—*qPH1a* (*bnlg1178‐bnlg1953*) and *qPH1b* (*umc2029‐phi011*)—to chromosome 1 using 294 maize RILs. Using two distinct populations—98 near‐isogenic introgression lines (Z3HBILs) in a Zong3 background and a backcross population derived from crossing Z3HBILs with recurrent parent Zong3—a PH QTL in bin1.03 was identified. This QTL was consistently detected across two growing seasons and showed repeated association with PH (Bai et al., 2010). Salvi et al. (2011) used B73 and Gaspé Flint as recipient and donor to construct a chromosome segment substitution line, and finally unearthed a primary QTL for PH in the interval of bin1.05‐1.06 (umc1395). These studies collectively reveal that chromosome 1 harbors numerous QTLs influencing maize PH, indicating that this region contains PH‐associated hotspots. However, most QTLs exhibit low repeatability across the studies, and only a few PH‐related genes have been successfully cloned to date. In this study, we identified a novel QTL *qPH_1.01_* on chromosome 1 using BSA, which could consistently decrease PH by 8–12 cm across multiple environments (2022HN, 2023YL, 2023HN) and explain 20%–30% of the phenotypic variation. Using a progeny‐based sequential fine‐mapping strategy, we narrowed down *qPH_1.01_* to an interval of ∼420 kb.

### 4.2 Exploring pleiotropic effects of the *qPH_1.01_*

Pleiotropic effects refer to the phenomenon in which a single gene or genetic locus affects multiple phenotypic traits (Solovieff et al., 2013). In plants, there exist multiple pleiotropic genes that often regulate core biological processes, thereby simultaneously influencing multiple important agronomic traits. In rice, the *IDEAL PLANT ARCHITECTURE1* (*IPA1*) locus, which encodes *OsSPL14* (SOUAMOSA PROMOTER BINDING PROTEIN‐LIKE 14), is a pivotal genetic determinant for improving tiller number, lodging resistance and grain yield (Jiao et al., 2010). In wheat, the *TaERF‐A1* encodes an AP2/ERF (APETALA2/ethylene responsive factor) transcription factor that regulates PH and spike length by regulating wheat stem elongation (R. Li et al., 2025). In maize, a CCT (CONSTANS, CO‐like, and TOC1) domain‐containing transcription regulator gene, *ZmCCT*, was reported to regulate flowering time and resistance to *Gibberella* stalk rot (C. Wang et al., 2017; Yang et al., 2010). In this study, we found that *qPH_1.01_* could concurrently regulate PH and EH of maize, and it not only reduces PH but also decreases EH by 4–6 cm. Although numerous PH and EH QTLs have been reported, few sites controlling both PH and EH have been excavated. Our discovery of *qPH_1.01_* represents a pleiotropic locus that coordinately regulates both PH and EH.

### 4.3 Exploring *qPH_1.01_* ′s regulatory mechanism on PH and EH

In the past 10 years, RNA‐seq has become an important technique for analyzing differential gene expression (Stark et al., 2019). Sun et al. discovered that mutations in the *ZmCYP90D1* gene displayed shorter PH and EH with 57.6% and 57.7% reductions, respectively, at the pollen shedding stage. Transcriptome sequencing analysis revealed that loss of function of *ZmCYP90D1* changed expression of hormone responsive genes, particularly BR‐responsive genes involved in cell cycle regulation, cell wall extension, and modification in maize (Sun et al., 2024). Fang et al. found that mutations in the *ZmWRKY92* reduced PH in maize. Transcriptome sequencing analysis revealed that the *ZmWRKY92* mutation caused substantial alterations in the expression of PH‐related genes. Further investigation established *ZmWRKY92* direct involvement in gibberellic acid (GA_3_) biosynthesis, with mutations significantly altering GA synthesis pathways (Fang et al., 2024). Zhu et al. discovered that knockout mutant of *ZmCPK39* resulted in a significant reduction in PH and EH. Transcriptomic analysis revealed that the downregulated genes in both *ZmCPK39* knockout lines and *ZmKnox2* mutants (encoding a ZmCPK39‐interacting protein) were significantly enriched in photosynthesis and carbon metabolic pathways. Furthermore, most of the downregulated genes were closely associated with auxin (M. Zhu et al., 2024). In this study, developing nodes and stalks at 40 days of a pair of near‐isogenic lines NIL‐*qPH_1.01PH6WC_* and NIL‐*qPH_1.01KA3321_* were collected for RNA‐seq analysis. GO analysis showed that the DEGs of nodes were enriched in the regulation of biosynthetic process, phosphate ion transport, cell differentiation, and anatomical structure morphogenesis (Figure 5C). The GO enrichment of stalks is enriched in hormone signaling pathway and DNA metabolic process (Figure 5E). In the enrichment of hormone signaling pathway DEGs, genes related to abscisic acid (ABA) were upregulated in NIL‐*qPH_1.01PH6WC_* in both the nodes and stalks; however, genes related to GA and BR were upregulated in NIL‐*qPH_1.01KA3321_* in both the nodes and stalks (Figure 6). We hypothesize that *qPH_1.01_* affects PH and EH by regulating the expression of hormone‐related genes, in particular ABA‐, BR‐, and GA‐responsive genes.

### 4.4 Dominant and recessive analysis of *qPH_1.01_* and *qPH_2.05_*

QTL effect validation (Figure 3) revealed distinct genetic architectures in terms of *qPH_1.01_* and *qPH_2.05_*. For *qPH_1.01_*, PH and EH differed significantly between *qPH_1.01PH6WC/KA3321_* heterozygotes and *qPH_1.01PH6WC/PH6WC_* homozygotes, but not between *qPH_1.01PH6WC/KA3321_* heterozygotes and *qPH_1.01KA3321/KA3321_* homozygotes, confirming its dominant effect (Figure 3A,C). For *qPH_2.05_*, PH differed significantly between *qPH_2.05PH6WC/KA3321_* heterozygotes and *qPH_2.05KA3321/KA3321_* homozygotes, but not between *qPH_2.05PH6WC/KA3321_* heterozygotes and *qPH_2.05PH6WC/PH6WC_* homozygotes, demonstrating recessive inheritance. Despite the cloning of several PH genes in maize, translating these findings into robust breeding outcomes continues to pose significant challenges (W. Wang et al., 2023). The difficulty likely stems from the fact that most PH genes are dominant for tallness and thus difficult to utilize, coupled with the limited number of dominant PH genes identified. In this study, we identified a dominant QTL, *qPH_1.01_*, that simultaneously reduced both PH and EH, demonstrating considerable promise for maize breeding applications.

### 4.5 Applications in maize breeding

PH is an important agronomic trait that affects high‐density tolerance and lodging resistance. Reducing PH and increasing planting density can significantly increase maize yield. Although a large number of QTLs and genes controlling PH have been excavated and cloned, most of them negatively impact maize yield due to excessive dwarfing or poor agronomic traits, ultimately compromising their breeding value. Thus, the discovery and identification of dwarf resources and key genes that reduce both PH and EH without yield penalties are vital to enable the development of maize cultivars suited for dense planting and mechanical harvesting. In the present study, we used an inbred line KA3321, to locate QTLs by pairing with PH6WC. The QTL on *qPH_1.01_* showed consistent and significant effects for reducing both PH and EH without affecting grain traits (Figure S2). The NILs containing the KA3321 allele could consistently decrease PH by 8–12 cm and EH by 4–6 cm than the PH6WC allele. Using a progeny‐based sequential fine‐mapping strategy, we narrowed down *qPH_1.01_* to an interval of ∼420 kb. Based on these results, the closely linked marker Ks_5.8 can be used in marker‐assisted selection (MAS) for reducing PH and EH in maize. To examine whether *qPH_1.01_* can consistently reduce PH and EH across different genetic backgrounds, we will utilize previously developed NILs carrying homozygous PH6WC and KA3321 alleles to simultaneously improve multiple inbred lines through MAS. Following multiple generations of backcrossing and selfing, we will compare PH and EH in the homozygous improved materials to determine: (1) if the KA3321‐derived allele maintains its PH and EH‐reducing effect across different inbred genetic backgrounds and (2) quantify the magnitude of its genetic effects. Although *qPH_1.01_* acts as a dominant QTL for reducing both PH and EH, the strong heterosis for PH in commercial hybrid maize often leads to increased PH through genetic interactions between parental genomes. To fully harness this QTL in breeding, we are implementing a dual‐parent improved strategy targeting both male and female lines. Overall, our findings demonstrate that *qPH_1.01_* is a major pleiotropic locus controlling PH and EH, and it can play a significant role in maize improvement.

## 5 CONCLUSION

In this study, we developed an F_2_ population by crossing the tall maize inbred line PH6WC with the short maize inbred line KA3321 to identify QTLs associated with PH. Utilizing BSA‐seq and field identification phenotypic data on the PH phenotype of a population of 617 F_2_ plants, a major dominant QTL, *qPH_1.01_*, on chromosome 1 was detected. Our findings indicate that *qPH_1.01_* is a stable QTL for PH and EH, with the allele from KA3321 reducing PH by 8–12 cm and EH by 4–6 cm, respectively. Through years of fine‐mapping, the *qPH_1.01_* region was mapped between molecular markers Ks_5.6 and Ks_6.0, with a physical distance of about 420 kb. Transcriptome data suggest that *qPH_1.01_* may affect PH and EH of maize by regulating the expression of hormone‐related genes. In the future, further fine‐mapping is essential to pinpoint the single candidate gene by generating and screening new recombinants within the mapped *qPH_1.01_* interval.

## AUTHOR CONTRIBUTIONS

**Chuan Chen**: Investigation; validation; writing—original draft; writing—review and editing. **Shaoqi Yang**: Investigation; validation; writing—original draft. **Ruiyu Zhang**: Data curation; formal analysis; writing—review and editing. **Yuan Tian**: Data curation; investigation. **Haotian Zhang**: Project administration. **Yapeng Wang**: Project administration. **Hongwei Zhang**: Writing—review and editing. **Zhe Wang**: Funding acquisition; methodology; supervision; writing—review and editing. **Qin Yang**: Conceptualization; data curation; funding acquisition; investigation; supervision; writing—review and editing.

## CONFLICT OF INTEREST STATEMENT

The authors declare no conflicts of interest.

## Supporting information




**Supplement FIGURE 1** BSA‐Seq data analysis with three different angorithem, the red lines show the threshold of each algorithms.
(A) Genomic distributions of total SNPs.
(B) Genomic distributions of sSNP/totalSNP ratios.
(C) Genomic distributions of G‐statistic values.
(D) Genomic distributions of ΔSNP‐index (Allele frequency) values. The red lines/curves are the thresholds.
**Supplement FIGURE 2** Grain‐related traits in the *qPH_1.01_* NILs.
A: Kernel size of NIL*‐qPH_1.01PH6WC_* and NIL*‐qPH_1.01KA3321_*. Scale bar = 1 cm.
B: Kernel length of NIL*‐qPH_1.01PH6WC_* and NIL*‐qPH_1.01KA3321_* (ns means no significant difference).
C: Kernel length of NIL*‐qPH_1.01PH6WC_* and NIL*‐qPH_1.01KA3321_* (ns means no significant difference).
D: Mature hundred‐ kernel weight of NIL*‐qPH_1.01PH6WC_* and NIL*‐qPH_1.01KA3321_* (ns means no significant difference).

**Table S1**. Primers used in this study.
**Table S2**. RNA‐seq data statistics.
**Table S3**. Effect validation of homozygous vs. heterozygous genotypes.
**Table S4**. Differentially expressed genes in nodes samples.
**Table S5**. Differentially expressed genes in stalks samples.
**Table S6**. GO enrichment results of differentially expressed genes in nodes samples.
**Table S7**. GO enrichment results of differentially expressed genes in stalks samples.

## Data Availability

RNA‐seq raw sequencing data were uploaded to the SRA database with accession PRJNA1287073. The data that support the findings of this study are available from the corresponding author upon reasonable request.
