# Supplementary material for: qPH1.01 , a dominant QTL that contributes to plant height and ear height in maize
Source: Plant Genome. 2025 Nov 20;18(4):e70157. doi: 10.1002/tpg2.70157 (PMC12635436; doi:10.1002/tpg2.70157)
Supplement: Supplementary file 1 [file TPG2-18-e70157-s002.docx]

**Supplement FIGURE 1** BSA-Seq data analysis with three different angorithem, the red lines show the threshold of each algorithms.

(A) Genomic distributions of total SNPs.

(B) Genomic distributions of sSNP/totalSNP ratios.

(C) Genomic distributions of G-statistic values.

(D) Genomic distributions of ΔSNP-index (Allele frequency) values. The red lines/curves are the thresholds.

**Supplement FIGURE 2** Grain-related traits in the *qPH_1.01_* NILs.

A: Kernel size of NIL*-qPH_1.01PH6WC_* and NIL*-qPH_1.01KA3321_*. Scale bar = 1 cm.

B: Kernel length of NIL*-qPH_1.01PH6WC_* and NIL*-qPH_1.01KA3321_* (ns means no significant difference).

C: Kernel length of NIL*-qPH_1.01PH6WC_* and NIL*-qPH_1.01KA3321_* (ns means no significant difference).

D: Mature hundred- kernel weight of NIL*-qPH_1.01PH6WC_* and NIL*-qPH_1.01KA3321_* (ns means no significant difference).
