# Supplementary material for: qPH1.01 , a dominant QTL that contributes to plant height and ear height in maize
Source: Plant Genome. 2025 Nov 20;18(4):e70157. doi: 10.1002/tpg2.70157 (PMC12635436; doi:10.1002/tpg2.70157)
Supplement: Supplementary file 1 — Supplement FIGURE 1 BSA‐Seq data analysis with three different angorithem, the red lines show the threshold of each algorithms. (A) Genomic distributions of total SNPs. (B) Genomic distributions of sSNP/totalSNP ratios. (C) Genomic distributions of G‐statistic values. (D) Genomic distributions of ΔSNP‐index (Allele frequency) values. The red lines/curves are the thresholds. Supplement FIGURE 2 Grain‐related traits in the qPH1.01 NILs. A: Kernel size of NIL‐qPH1.01PH6WC and NIL‐qPH1.01KA3321 . Scale bar = 1 cm. B: Kernel length of NIL‐qPH1.01PH6WC and NIL‐qPH1.01KA3321 (ns means no significant difference). C: Kernel length of NIL‐qPH1.01PH6WC and NIL‐qPH1.01KA3321 (ns means no significant difference). D: Mature hundred‐ kernel weight of NIL‐qPH1.01PH6WC and NIL‐qPH1.01KA3321 (ns means no significant difference). [file TPG2-18-e70157-s001.docx]

**
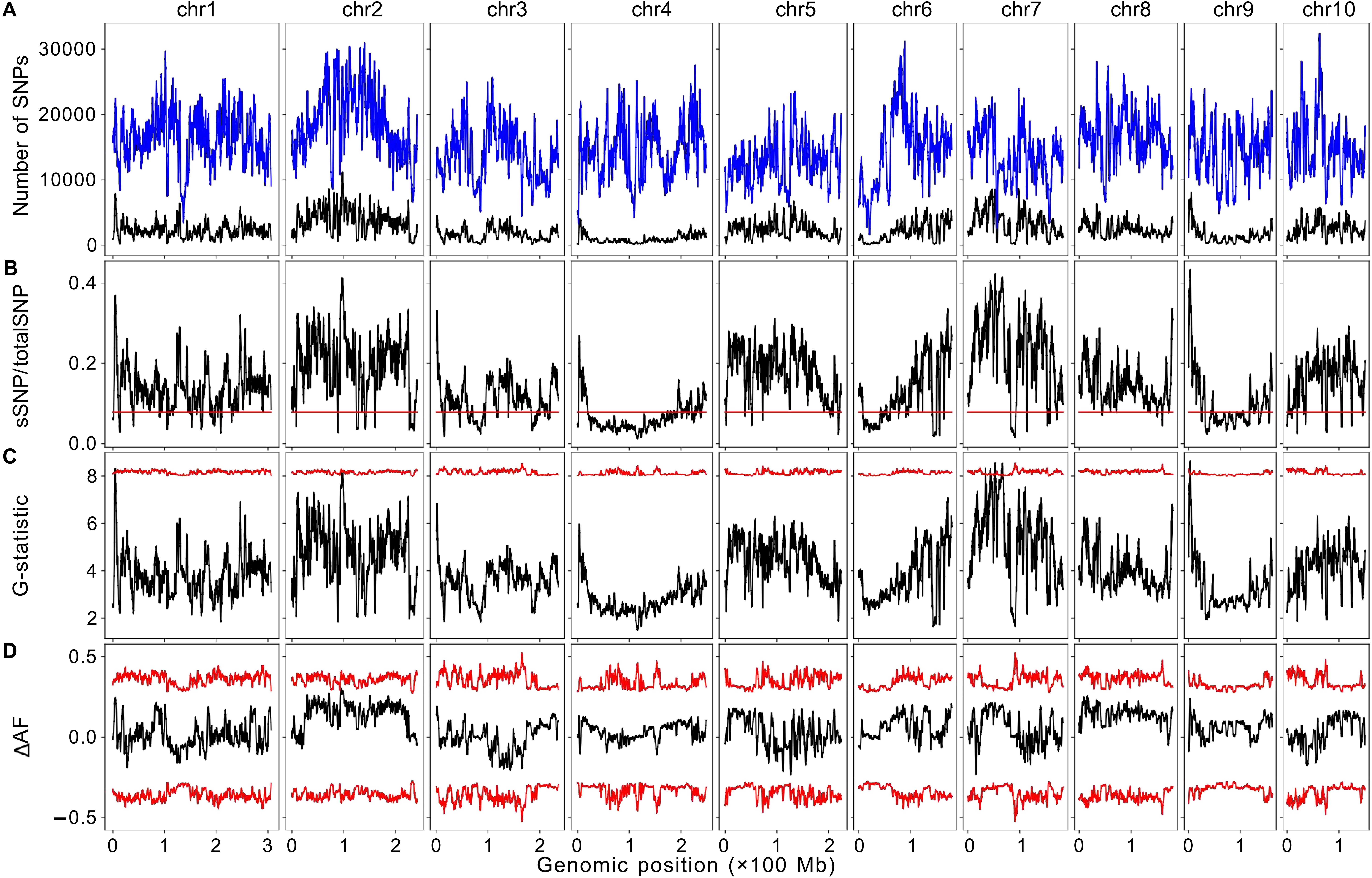
**

**Supplement FIGURE 1** BSA-Seq data analysis with three different angorithem, the red lines show the threshold of each algorithms.

(A) Genomic distributions of total SNPs.

(B) Genomic distributions of sSNP/totalSNP ratios.

(C) Genomic distributions of G-statistic values.

(D) Genomic distributions of ΔSNP-index (Allele frequency) values. The red lines/curves are the thresholds.


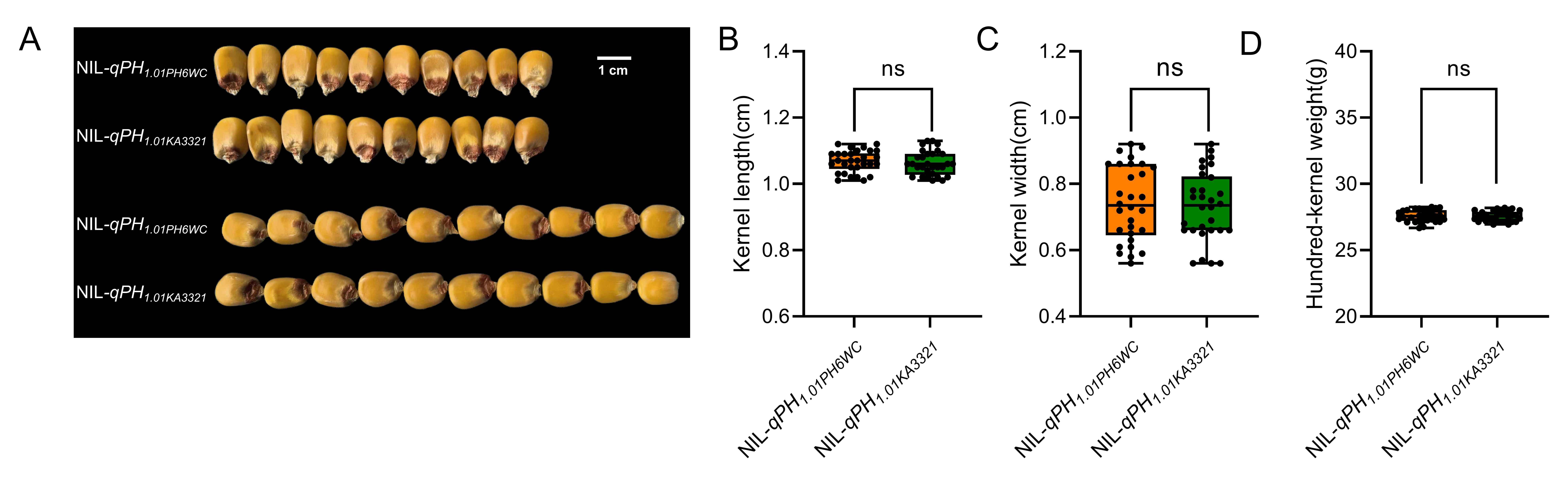


**Supplement FIGURE 2** Grain-related traits in the *qPH_1.01_* NILs.

A: Kernel size of NIL*-qPH_1.01PH6WC_* and NIL*-qPH_1.01KA3321_*. Scale bar = 1 cm.

B: Kernel length of NIL*-qPH_1.01PH6WC_* and NIL*-qPH_1.01KA3321_* (ns means no significant difference).

C: Kernel length of NIL*-qPH_1.01PH6WC_* and NIL*-qPH_1.01KA3321_* (ns means no significant difference).

D: Mature hundred- kernel weight of NIL*-qPH_1.01PH6WC_* and NIL*-qPH_1.01KA3321_* (ns means no significant difference).
